# *Genepleio* Software for Effective Estimation of Gene Pleiotropy from Protein Sequences

**DOI:** 10.1155/2015/269150

**Published:** 2015-01-05

**Authors:** Wenhai Chen, Dandan Chen, Ming Zhao, Yangyun Zou, Yanwu Zeng, Xun Gu

**Affiliations:** ^1^College of Mathematics & Information Science, Wenzhou University, Wenzhou, China; ^2^State Key Laboratory of Genetic Engineering and MOE Key Laboratory of Contemporary Anthropology, School of Life Sciences, Fudan University, Shanghai 200433, China; ^3^College of Life & Environmental Science, Wenzhou University, Wenzhou 325035, China; ^4^Shanghai Stem Cell Institute, Institutes of Medical Sciences, School of Medicine, Shanghai Jiao Tong University, Shanghai 200240, China; ^5^Department of Genetics, Developmental and Cell Biology, Iowa State University, Ames, IA 50011, USA

## Abstract

Though pleiotropy, which refers to the phenomenon of a gene affecting multiple traits, has long played a central role in genetics, development, and evolution, estimation of the number of pleiotropy components remains a hard mission to accomplish. In this paper, we report a newly developed software package, *Genepleio,* to estimate the effective gene pleiotropy from phylogenetic analysis of protein sequences. Since this estimate can be interpreted as the minimum pleiotropy of a gene, it is used to play a role of reference for many empirical pleiotropy measures. This work would facilitate our understanding of how gene pleiotropy affects the pattern of genotype-phenotype map and the consequence of organismal evolution.

## 1. Introduction

Understanding the role of gene pleiotropy in the map from genotypes to phenotypes has been one of the central topics for biologists, which refers to the phenomenon of a gene affecting multiple traits As a major measure for the functional importance of a gene, this concept has played a fundamental role in genetics, development, and evolution (see [1–3] for recent reviews and comments). However, the degree of gene pleiotropy remains largely unknown. Historically, proposed the concept of universal pleiotropy; that is, a single mutation can potentially affect all phenotypic traits. Though Fisher's model has been widely used as a theoretical basis for exploring the evolutionary interplay between the genotype and phenotype, the notion of universal pleiotropy has not been well tested.

Indeed, compared with the wide availability of genomics data, the whole-range phenotype recourses, or “phenomics,” are highly limited. Nevertheless, recent advances have been able to bring high throughput data to bear on the nature and extent of pleiotropy [4–6]. These experiments showed that the number of phenotypic traits that may be affected by a gene may be actually limited implying the role of modularity in shaping the degree of gene pleiotropy. Many controversial issues such as the problem of arbitrary number of correlated traits may directly affect the count of phenotypes that are predicted to be affected by a gene. On the other hand, a new approach has emerged in the past decade, to estimate the gene pleiotropy from genetics or sequence data, rather than from the affected phenotypes [7–13] (Chen et al., 2013). In particular, Gu [8] developed a practically feasible approach. It proposed that molecular evolution of a gene occurs in a multidimensional space corresponding to the same canonical number of molecular phenotypes. Random mutations of the gene could affect these molecular phenotypes constrained by the stabilizing selection. Moreover, Gu [8] developed a statistical method to estimate the “effective pleiotropy” (*K*_*e*_) of a gene from the multiple sequence alignment of protein sequences. Most genes have *K*_*e*_ in the range between 1 and 20 [11], with the medium of *K*_*e*_ = 6.5 of these estimates that is comparable to some empirical pleiotropy measures [1, 3]. Yet the relationship between these two approaches remains complex. As the degree of gene pleiotropy is a basic parameter for understanding the complexity of genotype-phenotype map, to facilitate this line of research we develop a software package* Genepleio* (freely available at http://www.xungulab.com) to estimate the effective gene pleiotropy from the protein sequences.

## 2. Material and Methods

### 2.1. Sequences

Three groups of datasets include eight vertebrates, twelve fruit flies, and seven yeast species. Each dataset contains 300 random selected orthologous sets. Eight vertebrates are fugu, zebrafish, xenopus, chicken, dog, cow, mouse, and human. Twelve* Drosophila* species are* D. melanogaster, D. pseudoobscura, D. sechellia*,* D*.* simulans*,* D. yakuba*,* D. erecta*,* D*.* ananassae*,* D*.* persimilis*,* D*.* willistoni*,* D. mojavensis, D*.* virilis,* and* D*.* grimshawi. *Seven yeast species are* Candida glabrata, Debaryomyces hansenii, Kluyveromyces lactis, Saccharomyces bayanus, Yarrowia lipolytica, Saccharomyces cerevisiae, *and* Schizosaccharomyces pombe. *Vertebrate CDS and protein sequences were extracted from Ensmart, Drosophila CDS and protein sequences were extracted from FlyBase, and yeast CDS was extracted from ORNA Man's dataset (corresponding protein sequences were translated by Bioperl).

### 2.2. Sequences Alignment

Multiple protein sequence alignment for each orthologous group was obtained by ClustalW at default settings.

### 2.3. Estimation of *d*_*N*_/*d*_*S*_

The number of synonymous substitutions per synonymous site (*d*_*S*_) and the number of nonsynonymous substitutions per nonsynonymous site (*d*_*N*_) between human and mouse orthologs were calculated by CODEML of PAML package [14]. When calculating the variance of *d*_*N*_ and *d*_*S*_, we changed “getSE = 1” in CODEML control file; otherwise, we used the default CODEML parameters. We used the estimates of *d*_*N*_/*d*_*S*_ between human and mouse for vertebrates, those between* D*.* melanogaster (dmel) and D. sechellia (dsec) *for* Drosophila,* and those between* S. bayanus and S. cerevisiae* for yeasts, respectively.

## 3. Results and Discussion

### 3.1. Estimation of Effective Gene Pleiotropy

Gu [8] analyzed the pleiotropy model of molecular evolution under the following assumptions. (i) *K*-dimensional molecular phenotypes (**y**) of the gene are under Gaussian-like stabilizing selection, indicating a single fitness optima for multiple functions. Any deviation from the optima is under the purifying selection. (ii) The fitness optima of **y** may shift randomly during the course of evolution, according to a multivariate normal distribution. It generates the process of* microadaptation* that could be caused by the external (environmental) or internal (physiological) perturbations or the functional compensation for the previously fixed slightly deleterious mutation. (iii) And the distribution of mutational effects, *p*(**y**), follows a multivariate normal distribution.

**Figure 1:**
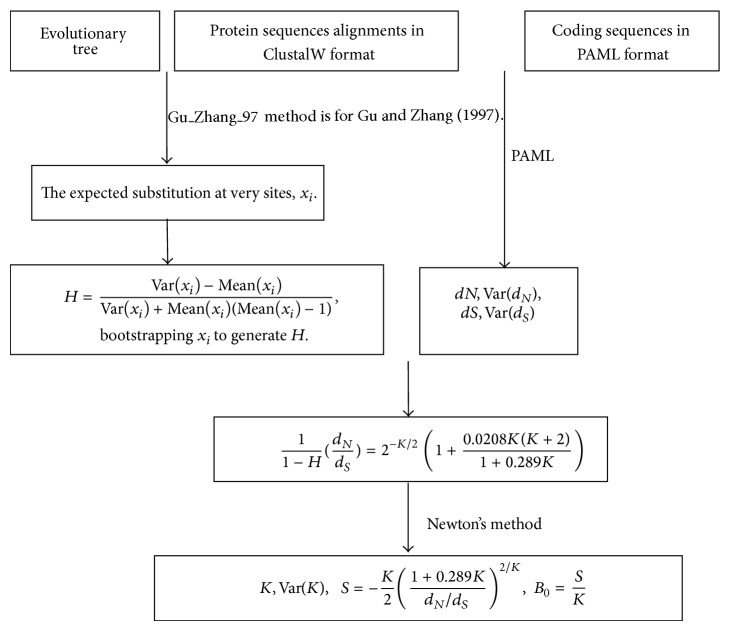
A flow chart to outline the computational pipeline implemented in software* Genepleio*.

The estimation procedure implemented in the software* Genepleio* is summarized in Figure 1. We address several key issues concisely to help in understanding how the software works. One may see Gu [8], Su et al. [11], and Gu (2014) for technical details.

#### 3.1.1. Calculation of *H*-Measure

Calculation of *H* is the key step to estimate *K*_*e*_. Biologically, *H* measures the strength of rate variation among sites: *H* = 0 when var(*λ*) = 0, and *H* = 1 when var(*λ*) = *∞*. After the gene phylogeny is given or inferred by the NJ option, the software implemented the methods of Gu and Zhang [15] to infer the number of amino acid changes along the phylogeny at each site, after correcting the multiple hits. The next step is to calculate the mean (*M*) and variance (*V*) of the estimated number of changes over sites. Under the Poisson-based evolutionary model, *H* can be estimated by *H* = (*V* − *M*)/[*V* + *M*(*M* − 1)].

#### 3.1.2. Estimation of Effective Gene Pleiotropy (*K*_*e*_)

*Genepleio* has implemented the following procedure to estimate *K*. (i) Calculate the *d*_*N*_/*d*_*S*_ ratio (the ratio of nonsynonymous to synonymous rates) used as an empirical measure for the mean sequence conservation. (ii) Calculate the *g*-function *g* = *d*_*N*_/*d*_*S*_/(1 − *H*). (iii) And the effective gene pleiotropy (*K*_*e*_) can be estimated by numerically solving the following equation:

(1) $$\begin{array}{c} \frac{d_{N}/d_{S}}{1-H}=2^{-K_{e}/2}\left[1+\phi \left(K_{e}\right)\right], \end{array}$$

where *ϕ*(*K*_*e*_) = 0.0208*K*_*e*_(*K*_*e*_ + 2)/(1 + 0.289*K*_*e*_).

#### 3.1.3. Estimation of Selection Intensities

There are two types of selection intensity measures. The first one is the (overall) selection intensity of the gene under study, *S*, for the overall strength of purifying selection imposed on the protein sequence; the negative sign indicates the negative (purifying) selection. The second one is the baseline selection intensity, *B*_0_, which is a scaled measure for the contribution of a single pleiotropy component. The relationship between *B*_0_ and *S* is *S* = −*K* × *B*_0_. When *K*_*e*_ is obtained, the software estimates the effective selection intensity *S*_*e*_ for *S* and the effective baseline selection intensity (*B*_*e*_) for the baseline selection intensity *B*_0_.

#### 3.1.4. Calculation of Sampling Variance of *K*_*e*_

The sampling variance of *K*_*e*_ can be approximately calculated by the delta method. Numerical analysis of (1) found that the following formula is accurate enough in practice:

(2) Var⁡Ke≈11.037Var⁡dN/dSdN/dS2+Var⁡H1−H2≈11.037Var⁡dNdN2+Var⁡dSdS2+Var⁡H1−H2.

In (2), Var⁡(*d*_*N*_/*d*_*S*_) can be estimated by the delta method so that  Var⁡(*d*_*N*_/*d*_*S*_) ≈ Var⁡(*d*_*N*_)/(*d*_*N*_)^2^ + (*d*_*N*_)^2^∗Var⁡(*d*_*S*_)/(*d*_*S*_)^4^, where Var⁡(*d*_*N*_), Var⁡(*d*_*S*_) are the variances of *d*_*N*_ and *d*_*S*_, respectively. The sampling variance *H* is difficult to compute analytically.* Genepleio* has implemented a bootstrapping approach to calculate the sampling variance of *H*, Var⁡(*H*), whereas sampling variances of *d*_*N*_ and *d*_*S*_, Var⁡(*d*_*N*_) and Var⁡(*d*_*S*_), depend on the users' input; their default values are set to be zero. Using this method, we can bootstrap 100 times within 1~2 minutes.

We use triosephosphate isomerase gene (TPI1, SWISSPROT P60174) for illustration. (i) Infer the phylogenetic tree from the multiple alignment of vertebrate homologous TPI1 protein sequences (human, mouse, dog, cow, chicken, xenopus, fugu, and zebrafish), which is consistent with the known vertebrate phylogeny. (ii) Estimate *d*_*N*_/*d*_*S*_ = 0.045 between the human and mouse genes by the likelihood method using PAML. (iii) Estimate *H*-index for the rate variation among sites; *H* = 0.614 for TPI1 gene. (iv) And we estimated *K*_*e*_ = 7.29 and the mean selection intensity *S* = −11.65. Then, the baseline selection intensity is given by *B*_0_ = 11.65/7.29 ≈ 1.60.

The estimated effective gene pleiotropy varies among different treatments but the scale of variation is small. On the other hand, we found that when the number of changes at each site is estimated by the parsimony method without any correction, gene pleiotropy tends to be overestimated. At any rate, we conclude that these 5–10% estimation differences should not affect the general pattern about the degree of gene pleiotropy.

### 3.2. Biological Interpretation of *K*_*e*_

The key question is how one can acquire the number of pleiotropy components of a gene without biologically knowing each component (Su et al., 2010) [12, 16]. Gu (2014) [17] addressed this issue, showing that the method of Gu [8] actually aims to estimate the rank (*K*) of genotype-phenotype map. The main result can be concisely represented by the following simple formula: *K* = min⁡(*r*, *P*_min⁡_), where *P*_min⁡_ is the minimum pleiotropy among all legitimate pleiotropy measures and *r* is the rank of mutational effects. In short, the meaning of “*effective gene pleiotropy*” (*K*_*e*_) estimated by Gu-2007 method is as follows. (i) *K*_*e*_ is an estimate of *K* = min⁡(*r*, *P*_min⁡_), the rank of genotype-phenotype map. (ii) *K*_*e*_ is an estimate for the minimum pleiotropy *P*_min⁡_ only if *P*_min⁡_ < *r*. (iii) Gu-2007 method attempted to estimate the pleiotropy of amino acid sites, a conserved proxy to the true minimum pleiotropy. (iv) With a sufficiently large phylogeny such that the rank of mutational effect at an amino acid site is *r* → 19 (the number of amino acid types minus one), one can estimate *P*_min⁡_ in the range from 1 to 19 by this method. And (*v*) *K*_*e*_ is a conserved estimate of *K* because those pleiotropy components that have small effects on fitness would be effectively removed by the estimation procedure.

### 3.3. Software Overview

**Figure 2:**
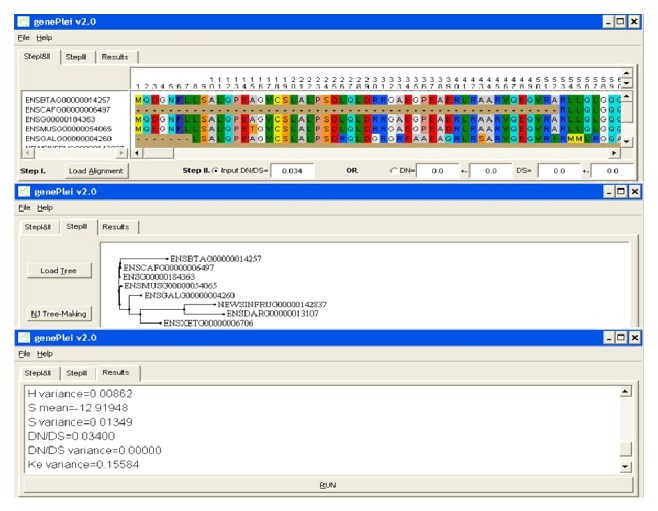
Screen illustration of the software* Genepleio*.

We have developed* Genepleio*, a GUI-based software package that estimates the effective gene pleiotropy from the phylogenetic sequence analysis of amino acids.* Genepleio* has three inputs. (i) Input the file of multisequence alignment (MSA) of protein sequences:* Genepleio* supports the alignment format of CLUSTALW. As required by the method, the multiple alignment file should contain at least four sequences with reasonably large sequence divergences between them. (ii) Input two values *d*_*N*_ (nonsynonymous distance) and *d*_*S*_ (synonymous distance). Several methods such as PAML can be used to obtain these estimates. We suggest choosing closely related sequences, say, *d*_*S*_ < 1, to avoid large sampling variance when calculating the *d*_*N*_/*d*_*S*_ ratio. Note that the *d*_*N*_/*d*_*S*_ ratio should be less than 1; otherwise, the gene may not be suitable to do this type of analysis. (iii) Input the tree file in the Phylip format. Alternatively, one may use the neighbor-joining (NJ) method implemented in the software to infer the gene phylogeny. As illustrated in Figure 2, the interface of* Genepleio* includes three main tab pages: the first page is for the MSA input and *d*_*N*_/*d*_*S*_ values, the second page is for the input or the inference of the phylogenetic tree, and the third page will output the results of estimation.

We have conducted a preliminary analysis and found that the 75% quantile of estimated *K*_*e*_ is typically within *K*_*e*_ ± 2, suggesting that *K*_*e*_ estimation as a measure of gene pleiotropy is statistically reliable. Besides, we notice that the contributions from Var⁡(*d*_*N*_) and Var⁡(*d*_*S*_) are nontrivial. In other words, the sampling variance of *K*_*e*_ would be severely underestimated if the user has no input for the sampling variances of *d*_*N*_ and *d*_*S*_.

There are some notices about usage of* Genepleio*. First, the multiple alignment file should contain more than four sequences; second, the *d*_*N*_/*d*_*S*_ value should be within (0,1); third, the sequences similarity >90% should be cautious because of the lack of statistical power; fourth, in order to shorten the time consumed, we do not give the mean of *K*_*e*_ value through bootstrapping in* Genepleio*. Nevertheless, according to much simulation, the mean value is close to the estimated *K*_*e*_ value, so we only give the estimated *K*_*e*_ value.

### 3.4. Computer Simulations

We have carried computer simulations to evaluate the software performance. We set *K* to vary from 1 to 20, with the fixed baseline selection intensity *B*_0_ = 0.5, 1.0, or 2.0, respectively. In particular, we consider three simulation models. (a) Independent-equal model: pleiotropy components are identical and independent of each other. (b) Independent-unequal model: pleiotropy components are independent of each other but have different strengths. (c) Random-matrix model: the strengths and correlations between pleiotropy components are randomly drawn from a specified random matrix model. Our main results are summarized in Table 1. In general, the estimation bias of *K*_*e*_ decreases with the increasing of the baseline selection intensity *B*_0_. For instance, in model (a), underestimation of *K*_*e*_ is considerable only when *B*_0_ is very small, say, 0.5 or less. The estimation bias becomes intermediate when *B*_0_ > 1 and becomes negligible when *B*_0_ > 3 (not shown). Moreover, the estimation bias of *K*_*e*_ may increase when the simulation model becomes more complex. Indeed, in model (c), *K*_*e*_ only describes the canonical number of pleiotropy components that could be much less than the number of pleiotropy components used in the simulation model.

### 3.5. Case Studies

**Figure 3:**
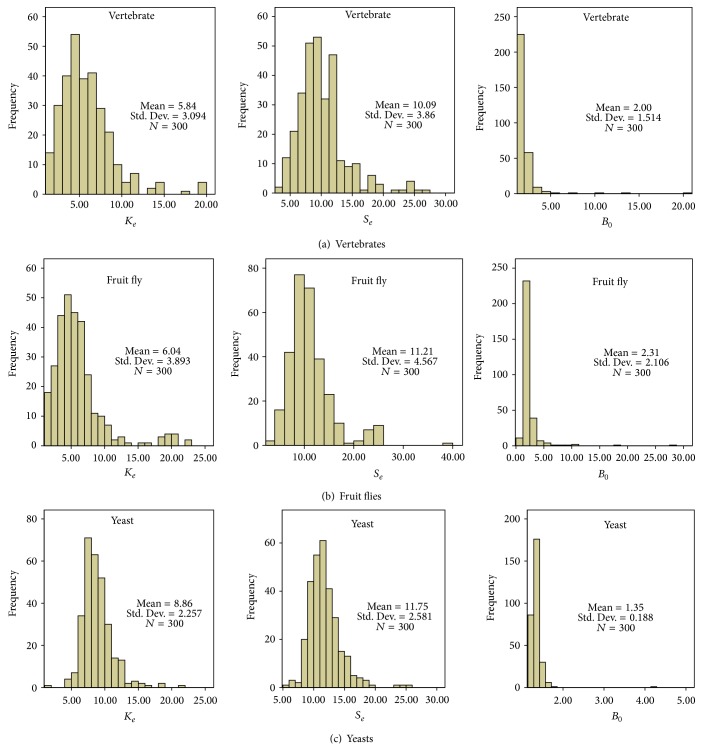
Estimation of *K*_*e*_ from three datasets: eight vertebrates, twelve fruit flies, or seven yeast species, respectively. Each dataset contains 300 random selected (one-to-one) orthologous sets.

To validate the performance of the newly developed software for the estimation of *K*_*e*_, we analyzed three datasets, each of which includes eight vertebrates, twelve fruit flies, or seven yeast species, respectively (Figure 3). Each dataset contains 300 random selected (one-to-one) orthologous sets. We calculated *K*_*e*_, *S*_*e*_ (selection intensity), and *B*_0_ (the baseline selection intensity). Our analysis shows that all *K*_*e*_ estimates are in a reasonable range with only a few outliners. Interestingly, the distribution of *K*_*e*_ estimates is similar across these distantly related species. The underlying reason to explain this similarity remains unclear. *K*_*e*_ is an important parameter for evolutionary analysis. Indeed, the square of coefficient correlation (*r* ^2^) between *K*_*e*_ and *S*_*e*_ is 0.64 in vertebrates, 0.94 in yeasts, and 0.55 in fruit flies, suggesting that gene pleiotropy may be an important evolutionary constraint in molecular evolution. In short, in this paper, we have reported a new software package* Genepleio* and demonstrated the steps of gene pleiotropy (*K*) estimation. We also examined the extent to which the statistical properties of *d*_*N*_/*d*_*S*_ and *H* affect the estimation efficiency of *K* and *S*. Comparison among three different groups of species validates the stability of *K* estimation procedure.

## Figures and Tables

**Table 1:** Simulation results of *K*_*e*_-estimation by Genepleio.

*K*	*B*_0_	*K*_*e*_ (model (a))	*K*_*e*_ (model (b))	*K*_*e*_ (model (c))
2	0.5	0.98 ± 0.03	0.94 ± 0.02	0.95 ± 0.02
4	0.5	1.98 ± 0.03	1.96 ± 0.03	1.58 ± 0.03
8	0.5	4.05 ± 0.05	3.67 ± 0.05	2.49 ± 0.03
12	0.5	6.16 ± 0.06	4.76 ± 0.06	3.89 ± 0.05
16	0.5	8.29 ± 0.13	6.65 ± 0.10	4.36 ± 0.06
2	1.0	1.34 ± 0.04	1.31 ± 0.04	1.31 ± 0.04
4	1.0	2.71 ± 0.06	2.13 ± 0.05	2.13 ± 0.05
8	1.0	5.44 ± 0.13	4.99 ± 0.10	3.26 ± 0.07
12	1.0	8.17 ± 0.29	6.56 ± 0.16	5.22 ± 0.10
16	1.0	10.9 ± 0.84	9.10 ± 0.37	5.85 ± 0.17
2	2.0	1.64 ± 0.06	1.60 ± 0.03	1.61 ± 0.06
4	2.0	3.28 ± 0.12	3.25 ± 0.05	2.61 ± 0.08
8	2.0	6.50 ± 0.40	6.12 ± 0.07	4.04 ± 0.15
12	2.0	9.67 ± 0.65	8.27 ± 0.07	6.59 ± 0.32
16	2.0	13.02 ± 0.67	11.30 ± 0.22	7.45 ± 0.46
